# Complete mitochondrial genome sequence of *Pareuchiloglanis kamengensis* in the Yarlung Zangbo River, Tibet

**DOI:** 10.1080/23802359.2019.1703576

**Published:** 2020-01-07

**Authors:** Qingzhi Ma, Lei Li, Xiaowan Lin, Hongyu Jin, Xing Jin, Bo Ma

**Affiliations:** a Heilongjiang River Fisheries Research Institute, Chinese Academy of Fishery Sciences, Harbin, P. R. China;; b College of Life Science and Technology, Harbin Normal University, Harbin, P. R. China;; c College of Fisheries and Life Science, Shanghai Ocean University, Shanghai, P. R. China

**Keywords:** *Pareuchiloglanis kamengensis*, mitochondrial genome, phylogenetic analysis

## Abstract

*Pareuchiloglanis kamengensis* belongs to the family of Sisoridae, *Pareuchiloglanis*. It is distributed in the Yarlung Zangbo River, the Irrawaddy River, the Nujiang River, and the Lancang River in southwestern China. In this study, we first published the complete mitochondrial genome sequence of *Pareuchiloglanis kamengensis*, which was 16,589 bp in length. This genome consists of 13 protein-coding genes (PCGs), 22 tRNA genes, two rRNA genes, and a non-coding A + T-rich region. The PCGs start with a traditional ATG except for COX1 and NAD3, which start with GTG and ATA instead, respectively, and end with stop codon TAA, TAG, TA, or a single T base. All tRNA have the typical clover-leaf structure. The phylogenetic tree of the whole mitogenome sequence is constructed by using neighbor-joining (NJ) method and the phylogenetic relationship among the family Sisoridae is further analyzed. We except to provide the theoretical basis for the further study of the phylogenetic relationship, taxonomic status, and conservation and management of genetic resources of Sisoridae catfishes.

## Introduction

*Pareuchiloglanis kamengensis* belongs to Sisoridae, *Pareuchiloglanis*, and is distributed in the Yarlung Zangbo River, the Irrawaddy River, the Nujiang River and the Lancang River in southwestern China. In Tibet, it is mainly distributed in the lower reaches of the Yarlung Zangbo River (Wu et al. 1981). In this study, *P. kamengensis* were collected from the Motuo section (29.18°N, 94.16°E) and were stored in the fish specimen room of Heilongjiang River Fisheries Research Institute, Chinese Academy of Fishery Sciences (specimen Accession number: XZ-1-001). We first published the complete mitochondrial genome of *P. kamengensis* and then analyzed the whole mitochondrial genome sequence.

The complete mitochondrial genome of *P. kamengensis* is a typical circular molecular of 16,589 bp in length and is deposited in GenBank (accession number: MN396886). It contains the typical set of 37 mitochondrial genes, including 13 protein-coding genes (PCGs), 22 tRNA, 2 rRNAs, and an A + T-rich region. The PCGs, tRNA gene, rRNA gene, and an A + T-rich region account for 69.56, 9.40, 15.98, and 5.38%, respectively. Among the 37 genes, *tRNA-Gln*, *tRNA-Ala*, *tRNA-Asn*, *tRNA-Cys*, *tRNA-Tyr*, *tRNA-Ser*, *tRNA-Glu*, *tRNA-Pro*, and *NAD6* genes are encoded in the H-chain, and the rest are in L-Chain coding. Thirteen PCGs of *P. kamengensis* contain three start codons (GTG, ATA, and GTG), except that the *COX1* and *NAD3* genes use GTG and ATA as the start codons, respectively, and all other PCGs use ATG as the start codon. There are four stop codons (TAA, TAG, TA-, and T–), in which TAG is the stop codon of *NAD1*, *COX1*, and *NAD6* genes. TAA is the stop codon of *NAD1*, *ATP8*, *ATP6*, *NAD4L*, and *NAD5* genes. *COX3* gene uses incomplete TA as a stop codon. *COX2*, *NAD3*, *NAD4*, and *Cyt b* genes use incomplete T as a stop codon. There are one or two nucleotides folding between two pairs of adjacent tRNAs in *P. kamengensis* tRNA genes (such as tRNA-IL and tRNA-Gln, tRNA-Thr and tRNA-Pro), which is also found in other catfishes (Lee et al. 2001). In this study, all 22 tRNA genes of *P. kamengensis* form the typical clover-leaf structure.

**Figure 1.**
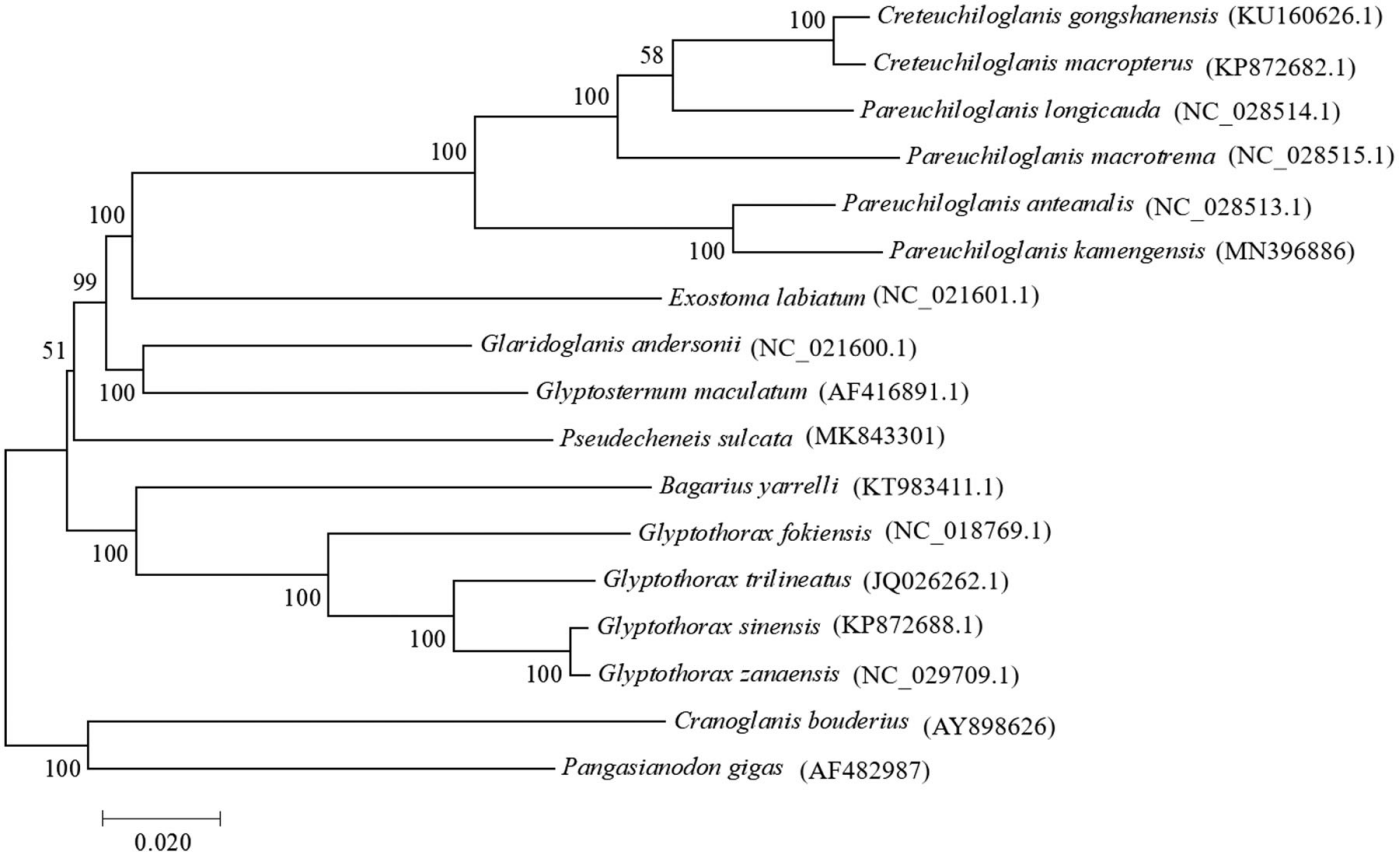
The phylogenetic tree (neighbor-joining) of *Pareuchiloglanis kamengensis* in this study and other 16 species based on the whole mitogenome sequence. Shown next to the nodes are bootstrap support values based on 1000 replicates.

In this study, *Cranoglanis bouderius* and *Pangasianodon gigas* are used as outgroups to build the phylogenetic tree of the whole mitogenome sequence by using the neighbor-joining (NJ) method (Figure 1). The monosyllabicity of Sisoridae catfishes is supported. The *P. kamengensis* in this study and the other three selected *Pareuchiloglanis* fishes also form a monophyletic group, which is consistent with Yu’s research results (Yu and He 2012). In addition, *Pseudecheneis* fishes have always been regarded as a group with an uncertain phylogenetic position in the study of Sisoridae catfishes. The results of this study show that the phylogenetic position of *Pseudecheneis* fishes is in the interior of the glyptosternoid fishes, which is consistent with the results of He (He 1996) and Guo et al (Guo et al. 2005, 2007).
